# Workforce and Staffing at 988 Suicide & Crisis Lifeline Centers

**DOI:** 10.1001/jamanetworkopen.2026.10789

**Published:** 2026-05-05

**Authors:** Samantha Matthews, Stephanie Brooks Holliday, Rachel Slover, James Liu, Joshua Breslau, Jonathan Purtle, Vikram Kilambi, Megan S. Schuler, Rajeev Ramchand, Jonathan Cantor, Ryan K. McBain

**Affiliations:** 1 RAND, Santa Monica, California; 2 RAND, Pittsburgh, Pennsylvania; 3 School of Global Public Health, New York University, New York, New York; 4 RAND, Arlington, Virginia; 5 RAND, Boston, Massachusetts; 6 Harvard Medical School, Boston, Massachusetts

## Abstract

**Importance:**

The 988 Suicide & Crisis Lifeline (998 Lifeline) receives millions of contacts annually. Adequate staffing of 988 Lifeline centers may be important for timely, high-quality service, but little information exists on current staffing levels or difficulties.

**Objectives:**

To describe 988 Lifeline center staffing and assess staffing-related difficulties.

**Design, Setting, and Participants:**

In this cross-sectional study, a survey was fielded between May 6 and July 25, 2025, to all 206 centers in the 988 Lifeline network in the US and territories. Eligible respondents were individuals in leadership positions (eg, executive directors, vice presidents).

**Main Outcomes and Measures:**

The survey measured staffing levels, shift coverage, modalities (telephone, text, and/or chat), operation of non-988 lines (eg, 211, local lines), proportions of paid and volunteer staff, remote and/or in-person work arrangements, and 4 domains of staffing difficulty: adequate staffing for the volume of contacts, acquiring funding to hire, recruiting staff, and retaining staff. Responses were linked with administrative data on location, presence of state 988 telecommunications fees, and subnetwork services (eg, national backup, Spanish language).

**Results:**

Leaders at 159 of the 206 centers completed the survey (77% response rate), 71% (102 of 144) reported that their center was understaffed, and 89% (141 of 159) indicated difficulty acquiring resources to hire. Leaders at centers offering remote work reported greater difficulty in obtaining these resources compared with centers without remote work (94% [89 of 95] vs 81% [50 of 62]; odds ratio [OR], 3.40; 95% CI, 1.21-9.68; *P* = .02) but less difficulty recruiting staff (76% [72 of 95] vs 89% [55 of 62]; OR, 0.39; 95% CI, 0.14-0.98; *P* = .04). Respondents from centers with all paid staff reported greater difficulty recruiting compared with centers using at least some volunteers (86% [102 of 118] vs 66% [27 of 41]; OR, 3.28; 95% CI, 1.42-7.60; *P* = .006). Leaders at centers handling only 988 contacts reported less difficulty retaining staff than those also handling non-988 lines (63% [17 of 27] vs 83% [109 of 132]; OR, 0.36; 95% CI, 0.14-0.92; *P* = .03). Wide 95% CIs indicate uncertainty in the magnitude of these results.

**Conclusions and Relevance:**

In this cross-sectional survey study of 988 Lifeline leaders, results suggested that most centers struggled to find resources to keep the center fully staffed. If staffing challenges persist, centers could face risks to staff well-being and service quality. Financing and operational strategies that support recruitment and retention will be critical to sustaining the quality and accessibility of 988 Lifeline centers.

## Key Points

**Question:**

What are the current staffing levels of 988 Lifeline centers, and what staffing challenges do these centers face?

**Findings:**

In this cross-sectional survey study using data from a national survey of 988 Lifeline centers, leaders from 77% of call centers across 47 US states and territories responded. A total of 71% of leaders reported that their center was understaffed; when asked about staffing-related difficulties, almost 90% of respondents reported difficulty with acquiring resources to hire.

**Meaning:**

This study suggests that 988 Lifeline center staffing shortages could be associated with operational demands, underscoring the importance of increased investments to bolster the 988 Lifeline workforce.

## Teaser

This cross-sectional survey study describes 988 Suicide & Crisis Lifeline center staffing and assesses staffing-related difficulties faced by these centers.

## Introduction

The US national crisis hotline, the 988 Suicide & Crisis Lifeline (988 Lifeline), launched in July 2022. Callers, texters, and chatters to 988 are connected to the 988 Lifeline, formerly known as the National Suicide Prevention Lifeline.^1^ The shift from a 1-800 telephone number to 988 aimed to increase accessibility of the hotline^1^ and extend beyond suicide prevention and include mental health crises more broadly.^2^ Since the launch of the 988 Lifeline, it has been contacted nearly 18 million times, 68% of which were calls, 18% were texts, and 15% were chats.^3^ Contacts have consistently grown, from 3.7 million in 2022 to 6.5 million in 2024.^3^

At the time of the 988 Lifeline’s launch, policymakers and advocates expressed concern about whether the existing hotline infrastructure would be sufficient.^4,5,6,7^ Jurisdictions differed substantially in terms of financial and human resources,^8^ and 988 Lifeline centers expected increases in contact volume.^2,9^ In addition to the increased federal investment to support the Lifeline after 988 implementation,^10^ states were encouraged to establish telecommunications fees to support 988 center costs and to finance crisis services,^2^ similar to fees that finance the 911 system.^11^ By October 2025, 12 states and 1 US territory had enacted legislation to fund the 988 Lifeline through such fees.^12^

Working for a crisis center is challenging. Counselors must keep calm under pressure, deliver support in circumstances where callers face systemic barriers to care, cope with occasionally abusive and inappropriate language,^12^ and adhere to standards for safety assessment procedures.^13^ The emotional demands of crisis line work can contribute to compassion fatigue and psychological distress, which are associated with functional impairment and increased turnover intentions.^13,14^ Counselors with lived experience of suicide report higher levels of stress, depression, burnout, and secondary trauma compared with colleagues without this risk factor.^15^ Among volunteers, 18% report moderate to high distress, 16% indicate they might leave their role, and 4% are likely to leave within 1 year.^14^ These challenges underscore the importance of workforce well-being.

Counselors are a mix of paid staff and volunteers and may work in person and/or remotely depending on center policies.^14,15^ Remote work has been proposed as a strategy to address workforce shortages^16^ and volunteers may help fill gaps in staffing; however, the number of hours that volunteers work appears important. In one multisite study, volunteer counselors who worked significantly fewer hours per week than paid staff had lower odds of collaboratively engaging callers with imminent suicide risk and higher odds of initiating an active rescue (ie, involuntary emergency service intervention for people at imminent risk of suicide).^16^ The study showed that higher weekly call hours, regardless of role, were linked with more engagement, less reliance on active rescue, and a higher likelihood of reducing imminent risk. These findings suggest both volunteer and paid staff can successfully support callers in reducing their suicide risk and preventing the need for an in-person response.

Currently, policymakers and administrators have limited information on staffing at 988 Lifeline centers.^5^ A 2022 survey found that many states reported crisis center workforce shortages, particularly among clinical social workers and peer specialists,^17^ but no data on full-time equivalent staff have confirmed this speculation.^18^ This paucity of data is problematic, as adequate staffing may reflect whether 988 Lifeline centers have sufficient funding to achieve their mandate.^19^ As in other workplaces, staffing levels could potentially affect answer rates, wait times, and overall performance. This study’s purpose is to quantify current levels of 988 Lifeline center staffing and levels of difficulty in adequately staffing these centers.

## Methods

Between May 6 and July 25, 2025, we fielded a survey to all 206 centers in the 988 Lifeline network (at the time of the survey) via an email from Vibrant Emotional Health (Vibrant) directed to crisis center directors or senior operational leaders (1 survey per center). Based on contact information provided by Vibrant and searches of call center websites, RAND conducted multiple follow-up contacts. We first contacted executive directors (or equivalent roles) and if we did not receive a response, we proceeded to reach out to other leaders in the organizational hierarchy. One leader at each 988 Lifeline center was invited to complete the 34-question survey, provided written or oral informed consent prior to participation, and was offered a $50 Amazon gift card for completion. If we received more than 1 response from a center, we retained the response from the most senior director. Study procedures and protocols were approved by RAND’s institutional review board. The study followed the American Association for Public Opinion Research (AAPOR) reporting guidelines for survey studies.

### Measures

Informed by previous work^20^ and other surveys related to the 988 Lifeline,^9^ this study’s survey asked respondents about the modalities offered (call, text, and/or chat), whether the center answered non-988 lines (eg, 211, 311, local crisis line), and, if so, the proportion of contacts reached through 988. The survey also included detailed staffing questions. These questions asked whether counselors were paid or volunteer staff, the proportion of paid counselors, the number of counselors working a typical shift, the total number of full-time equivalent counselors, the ideal number of additional full-time equivalent counselors needed for full staffing, and whether counselors are required to work in person or can work remotely. We matched each response with administrative data we had on each center, specifically, the location, location in a state with a 988 telecommunications fee, and subnetwork services (eg, national backup, Spanish language).

With regard to staffing difficulty, we measured 4 domains: adequate staffing for the volume of contacts received, acquiring funding to hire staff, recruiting staff, and retaining staff. These questions were asked on a 5-point Likert scale from “not difficult” to “very difficult.” The full survey can be found in the eAppendix in Supplement 1.

### Statistical Analysis

We calculated and reported descriptive statistics for all responses and conducted bivariate analyses to examine associations between center characteristics and staffing difficulties, using Fisher exact tests. We applied a unified test inversion approach based on the likelihood ratio test (exact2x2 package, tsmethod = “minlike”) to generate 95% CIs consistent with *P* values in Fisher exact tests.^21^ Center characteristics included location (US region); being in a state with a 988 telecommunications fee in effect at the time of the survey; serving as a national backup center (ie, routed contacts not answered locally); offering text and/or chat; answering non-988 lines; having all paid staff; and offering remote work. The dependent variables (each of the 4 staffing domains) were dichotomized for analysis. Responses indicating any level of difficulty were deemed as “having staffing difficulty” and coded as “1.” The response “not difficult” was considered “no staffing difficulty” and coded as “0.” This approach was selected due to the small sample size and the uneven distribution of response categories.

All statistical tests were conducted in R, version 4.5.1 (R Project for Statistical Computing) and used a 2-sided α level of .05. Missing and incomplete data for 6 survey questions were addressed via case-wise deletion. The proportion of missing data ranged from 1% to 10%, except for the item asking about the percentage of contacts from 988 vs other lines, which had 33% missing responses.

## Results

The survey was completed by leaders at 159 of 206 centers (77% of all 988 Lifeline centers at the time of the survey) located in 47 US states and territories. Roles included executive director, director, director of crisis services, and program manager. Responding centers spanned the US, located in the Northeast (34 of 159 [21%]), Midwest (42 of 159 [26%]), South (55 of 159 [35%]), and West (25 of 159 [16%]) (Table 1). Sixteen percent of centers (25 of 159) were located in a state with a 988 telecommunications fee in effect at the time of the survey. All but 2 respondents reported their center answered telephone calls. The 2 centers that did not answer calls handled only texts and chats. Overall, 50% of centers (80 of 159) answered texts and/or chats.

**Table 1.** Operational and Staffing Characteristics of 988 Suicide & Crisis Lifeline Centers

Center characteristic	No. (%) (N = 159)
988 Subnetwork service^a^	
Local only	119 (74.8)
National backup center	31 (19.5)
Disaster Distress Helpline	10 (6.3)
Spanish language	7 (4.4)
LGBTQ+ Line (ended July 2025)	7 (4.4)
Veterans Crisis Line	4 (2.5)
Region^b^	
Northeast	34 (21.4)
Midwest	42 (26.4)
South	55 (34.6)
West	25 (15.7)
State 988 fee in effect	
Yes	25 (15.7)
No	134 (84.3)
988 Modalities^a^	
Telephone	157 (98.7)
Text	66 (41.5)
Chat	65 (40.9)
Lines answered	
Non-988 lines	132 (83)
Only 988	27 (17)
Occupational status	
All paid staff	118 (74.2)
Mix of paid and volunteer staff	40 (25.2)
All volunteer staff	1 (<0.1)
Remote policy^c^	
Fully remote allowed	68 (43.3)
In person only	62 (39.5)
Hybrid (ie, remote and in person)	27 (17.2)

Abbreviation: LGBTQ+, lesbian, gay, bisexual, transgender, queer, and other sexual and gender minority.

^a^ Not mutually exclusive.

^b^ N = 156.

^c^ N = 157.

In addition to locally routed calls, some centers provided coverage for 988 Lifeline subnetwork and national backup service lines. Specifically, 31 centers served as part of the national backup network, handling 988 contacts not answered by local centers. Other centers responded to specialized lines, including the Disaster Distress Helpline (n = 10; available during disasters), the Spanish Language Line (n = 7; accessed by pressing 2 after calling 988) the LGBTQ+ (lesbian, gay, bisexual, transgender, queer, and other sexual and gender minority) Line (n = 7; previously accessed by pressing 3 but was discontinued in July 2025), and the Veterans Crisis Line (n = 4; accessed by pressing 1 after calling 988). Response rates did not differ significantly by subnetwork service.

In addition to handling 988 contacts, 132 respondents (83%) reported that their center answered other hotlines, including 211 (an informational line for community resources such as food, housing, and health care), 311 (a nonemergency government service line for issues such as potholes or noise complaints), or a local mental health line (Table 1). Counselors typically answered multiple lines; of these 132 centers, only 12 (9%) had counselors who answered only 988 calls on a given shift. Contacts via 988 accounted for a mean (SD) of 45% (29%) of all contacts at these centers.

Staffing levels varied widely, from 1 to 309 counselors (mean [SD], 34 [47]; median, 20). On a typical shift, centers reported a mean (SD) of 9 (16) counselors on duty, indicating a skewed distribution with a few centers reporting substantially larger teams. For example, 7 centers reported 50 or more counselors worked a typical shift. Almost all respondents reported their center (158 [99%]) employed paid personnel as counselors, with 74% (118 of 159) employing only paid personnel (Table 1). One-fourth of centers (40 of 159) had a mix of paid and volunteer counselors, with a mean (SD) of 75% (26%) of counselors being paid. With regard to work arrangements, 60% of centers (95 of 159) allowed remote work either full-time (43% [68 of 159]) or on a hybrid schedule (ie, a mix of remote and in person; 17% [27 of 159]). The other 40% of centers (62 of 159) had counselors work in person only. Table 1 displays center operational and staffing characteristics.

Staffing shortages were common among respondents. When asked their ideal staffing levels, only 29% (42 of 144) of respondents reported that their center was fully staffed, while 71% (102 of 144) of respondents reported that their center was understaffed.

The most frequently cited staffing challenge among survey respondents was acquiring resources to hire staff. Eighty-nine percent of respondents (141 of 159) reported that their center found it difficult to acquire such resources, with 20% (31 of 159) reporting this acquisition to be very difficult. Similarly, 87% (139 of 159) reported difficulty with adequately staffing for their contact volume, but only 9% (14 of 159) found this to be very difficult. About 80% of respondents reported that their centers found recruiting (81% [129 of 159]) and retaining (79% [126 of 159]) staff difficult. The Figure displays reported levels of difficulty across these staffing domains.

**Figure.**
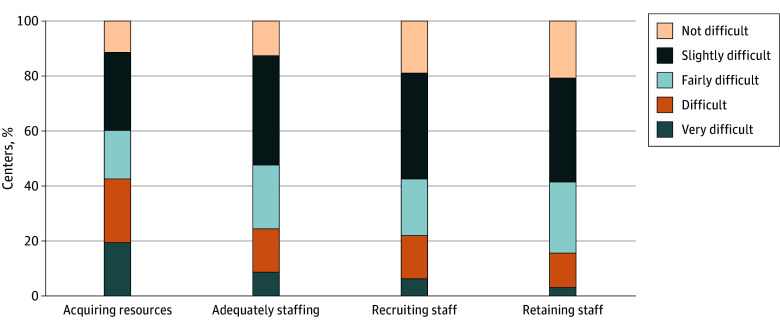
Bar Graph Showing Level of Difficulty 988 Lifeline Centers Face in Acquiring Resources to Hire (n = 159) Centers were prompted with: “For each of the following items, please indicate the level of difficulty your call center experiences with regard to staffing.”

Bivariate analyses indicated that leaders from centers allowing remote work had higher odds of reporting difficulty acquiring resources to hire compared with leaders of centers without remote work options (94% [89 of 95] vs 81% [50 of 62]; odds ratio [OR], 3.40; 95% CI, 1.21-9.68; *P* = .02) (Table 2). The wide 95% CI indicates uncertainty in the magnitude of the result. No other center characteristics were associated with reported difficulty acquiring resources to hire, and no characteristics were associated with reported difficulty adequately staffing for their contact volume. Table 2 displays all associations tested.

**Table 2.** Association Between 988 Suicide & Crisis Lifeline Center Characteristics and Reported Staffing Difficulties (N = 159)

Center characteristic	Acquiring resources to hire		Adequately staffing		Recruiting staff		Retaining staff	
	OR (95% CI)	*P* value	OR (95% CI)	*P* value	OR (95% CI)	*P* value	OR (95% CI)	*P* value
National backup center	0.59 (0.19-1.86)	.35	0.96 (0.30-3.37)	>.99	0.75 (0.29-2.18)	.61	1.45 (0.51-4.30)	.62
Call center region								
Northeast	1 [Reference]	NA	1 [Reference]	NA	1 [Reference]	NA	1 [Reference]	NA
Midwest	1.25 (0.27-6.59)	>.99	0.50 (0.09-2.32)	.33	0.33 (0.08-1.21)	.08	0.55 (0.14-2.04)	.38
South	1.33 (0.24-6.72)	.73	1.74 (0.42-7.20)	.52	2.42 (0.75-8.09)	.11	1.23 (0.38-3.87)	.79
West	1.52 (0.20-18.21)	>.99	5.03 (0.55-245.92)	.22	1.31 (0.35-5.24)	.77	0.97 (0.25-4.02)	>.99
State 988 fee in effect	0.92 (0.24-4.04)	>.99	3.94 (0.61-84.56)	>.99	0.92 (0.30-2.79)	>.99	0.80 (0.28-2.23)	.79
Answer text and/or chat	0.84 (0.31-2.37)	.81	1.07 (0.41-2.85)	>.99	0.66 (0.28-1.50)	.32	1.36 (0.63-3.08)	.44
Answer only 988 contacts	0.48 (0.15-1.54)	.19	0.79 (0.24-2.80)	.75	0.61 (0.22-1.80)	.29	0.36 (0.14-0.92)	.03
All paid staff	0.54 (0.13-1.98)	.57	0.69 (0.20-2.14)	.78	3.28 (1.42-7.60)	.006	1.90 (0.80-4.47)	.12
Remote work allowed	3.40 (1.21-9.68)	.02	1.25 (0.44-3.21)	.64	0.39 (0.14-0.98)	.04	0.81 (0.36-1.82)	.69

Abbreviations: NA, not applicable; OR, odds ratio.

With regard to recruitment, respondents from centers allowing remote work had lower odds of reporting difficulty compared with those from centers without remote work (76% [72 of 95] vs 89% [55 of 62]; OR, 0.39; 95% CI, 0.14-0.98; *P* = .04) (Table 2). Respondents of centers with all paid staff also had higher odds of reporting difficulty with recruitment compared with centers using some volunteers (86% [102 of 118] vs 66% [27 of 41]; OR, 3.28; 95% CI, 1.42-7.60; *P* = .006), but there is uncertainty in the magnitude of this result. No other characteristics were significantly associated with reported difficulty with recruitment. With regard to retaining staff, only 1 characteristic was statistically significant: centers answering only 988 contacts had lower odds of reporting retention difficulties (63% [17 of 27] vs 83% [109 of 132]; OR, 0.36; 95% CI, 0.14-0.92; *P* = .03) compared with centers also handling non-988 lines (Table 2).

## Discussion

To our knowledge, this is the first cross-sectional survey study of leaders of 988 Lifeline centers and their staffing capacity. Nearly three-quarters of responding centers’ leadership reported being understaffed, consistent with broader workforce shortages in behavioral health care. For example, in a 2024 National Association of State Mental Health Program Directors Research Institute report, 40 states reported workforce shortages in their behavioral health crisis systems.^22^ Relatedly, a 2024 survey of 988 Lifeline center directors and crisis systems leaders found only 23% strongly agreed that crisis service provider salaries have been sufficient to retain quality staff and minimize turnover.^9^ In our study, the most frequently reported staffing challenge was acquiring adequate funding and resources to hire. This finding aligns with the aforementioned 2024 study, which found only 10% of respondents reported sufficient funding to meet demand for 988.^9^ Future research should include qualitative methods to better understand factors contributing to these funding challenges.

Respondents of 988 Lifeline centers with remote work options reported less difficulty recruiting staff than those with only in-person workers, suggesting that flexible arrangements may broaden the applicant pool and make positions more appealing. This finding is consistent with research showing remote work can support job satisfaction and decrease stress.^23^ However, these centers also reported greater challenges obtaining resources to hire, which may reflect structural or funding limitations with remote operations or a shift toward offering remote work due to resource constraints. Remote work may alleviate workforce shortages but could require tailored funding approaches to sustain them, such as investments in telework technology, remote supervision and training, and mechanisms to recruit staff from outside a center’s geographic area or state. Factors other than remote work alone could underlie this association, such as agency regulations, location, or organizational structure. Future research should explore these associations, such as by examining potential salary differences influencing remote work in 988 Lifeline centers.

Respondents from centers with all paid staff reported greater recruitment challenges than those with some volunteers. This may be due to centers with all paid staff facing greater financial burdens when recruiting. Incorporating volunteers may provide a more flexible source of workers and may help centers fill staffing gaps. Other factors could influence recruitment challenges, such as the diversity of communities from which centers recruit staff. Future research should examine these recruitment pools.

Respondents from centers answering only 988 contacts rather than multiple service lines reported fewer retention challenges, suggesting that multiservice line models may affect workload, role complexity, or overall organizational stability and resource needs, while centers serving only contacts from the 988 line may benefit from a distinct mission, organizational identity, or supportive infrastructure. This finding also suggests staffing challenges may be associated not only with 988 but also with broader hotline services. Additional information on how multiple lines are staffed and organized within centers is needed.

This study’s findings reinforce the concern that the sizeable increase in the number of contacts to 988 may come into conflict with a strained crisis workforce.^24,25,26^ Future research is needed to determine if staffing shortfalls have implications for 988 Lifeline service quality, response times, and staff well-being. Evidence from nursing research suggests inadequate staffing contributes to increased staff stress and burnout,^27^ higher turnover,^28^ longer wait times,^29^ and adverse patient outcomes.^29^ One related but dated study available on crisis line volunteers found that turnover was the most frequently cited factor contributing to burnout.^30^

Previous research suggests that the mechanisms to support and retain crisis center staff include competitive wages, remote work, shift differential payments in response to increased demand (such as on nights and weekends), promoting counselors to mentor positions, and offering part-time positions.^31^ Thus, this surveys’ results on remote work are notable. Leaders of 988 Lifeline centers who offered remote work reported fewer recruitment challenges but greater difficulty securing resources to hire. This finding aligns with existing literature showing remote workers earned higher wages than on-site workers and experienced greater wage growth during the COVID-19 pandemic.^32^ As a result, sustaining recruitment of hybrid or fully remote workers may require new financing models to support staffing. Payment for such adjustments may require new funding mechanisms to ensure consistent, sustainable funding.^31^

### Limitations

This study has several important limitations. First, despite a high response rate (77%), the survey relied on self-reported data from a subset of 988 Lifeline center leaders, which may have introduced response bias affecting the accuracy of information provided. For example, leaders may differ in how they define adequate staffing levels or resources to hire. Although participating centers represented most of the national network, nonparticipation introduces uncertainty about the representativeness of these findings. Second, responses reflect staffing conditions only at the time of the survey, which coincided with notable changes to the 988 Lifeline—including policy updates, the discontinuation of the LGBTQ+ line, and increasing demand. These developments may have influenced respondents’ perceptions, limiting generalizability but also providing important context for understanding workforce experiences during this transition period. Third, unmeasured state-level and regional-level factors may have influenced results. Fourth, because the analyses were based on a finite number of centers, some subsamples were small and uneven, which may have contributed to wide 95% CIs and limited precision. This also led us to dichotomize outcome measures in a few instances, which reduced resolution of results to generate larger grouped response categories. Given this study’s exploratory nature, we did not apply corrections for multiple comparisons, which increases the probability of a type I error. Fifth, as a cross-sectional study, this analysis measures associations only; no findings should be interpreted as causal.

That said, this study contributes critical insights to the limited literature on the 988 Lifeline workforce,^17^ offering the first national, center-level estimates of staffing levels and challenges. Prior research identified concerns about preparedness before 988 Lifeline’s launch,^8,33^ and concerns about sustainable funding have continued,^2,9^ which have been exacerbated by the absence of detailed workforce data. This study’s findings reinforce the need for policymakers to implement system-level solutions that strengthen the 988 Lifeline’s responsiveness and improve crisis outcomes.

## Conclusions

The launch of the 988 Lifeline marked an important moment in US behavioral crisis response. Its success, however, depends on the stability and strength of its workforce. This cross-sectional survey study’s findings suggest that center leaders face staffing shortages and challenges in recruiting and retaining counselors, with financing emerging as a key barrier. Continued efforts to implement and evaluate sustainable funding models, improve operational efficiencies, and develop innovative recruitment strategies to strengthen the 988 Lifeline workforce are needed to sustain the accessibility and quality of crisis care.

## Notes

Supplement 1. **eAppendix.** Survey Instrument

Supplement 2.
Data Sharing Statement
