# Error in Funding/Support

**DOI:** 10.1001/jamanetworkopen.2026.22812

**Published:** 2026-06-17

In the Original Investigation titled “Workforce and Staffing at 988 Suicide & Crisis Lifeline Centers,”^1^ published May 5, 2026, the grant number in Funding/Support was incorrect. It should be grant 5R01MH135034, not 1R01MH132551. This article has been corrected.^1^
