# Supplementary material for: Workforce and Staffing at 988 Suicide & Crisis Lifeline Centers
Source: JAMA Netw Open. 2026 May 5;9(5):e2610789. doi: 10.1001/jamanetworkopen.2026.10789 (PMC13147195; doi:10.1001/jamanetworkopen.2026.10789)
Supplement: Supplement 1. — eAppendix. Survey Instrument [file jamanetwopen-e2610789-s001.pdf]

Supplementary Online Content

Matthews S, Holliday SB, Slover R, et al. Workforce and staffing at 988 Suicide & Crisis Lifeline centers. *JAMA Netw Open.* 2026;9(5):e2610789.
doi:10.1001/jamanetworkopen.2026.10789

eAppendix. Survey Instrument

This supplementary material has been provided by the authors to give readers additional information about their work.

eAppendix. Survey Instrument

CRISIS CALL CENTER SURVEY: CAPACITY AND CAPABILITIES

INTRODUCTION

WHO: This survey is being completed by leaders at 988 and other crisis call centers around the country and is led by RAND, a non-profit, non-governmental research organization.

WHAT: This survey gathers information on your call center's staffing and service capabilities. Your responses are completely confidential—this is NOT a government performance review. Instead, we're aiming to highlight nationwide resource needs, helping inform funders and policymakers about your center's requirements.

HOW LONG: The survey will take about **10 minutes** to complete. You can start the survey and complete it later if you need to seek out information that you don't have on hand.

CONFIDENTIALITY: To preserve confidentiality of respondents, your name will not be listed in any analyses or presentations of call center information. We plan to share results from call centers throughout the United States aggregated at county, census region, and state levels. We believe this information is important so that funders and policymakers understand your resource needs.

VOLUNTARINESS: Participation is voluntary, and you can stop at any time without consequence to you or your organization.

COMPENSATION: You will be offered a **$50 gift card to Amazon** for completing the survey, which you do not need to accept.

- o Yes, I would like to continue.
- o No, thank you. I prefer not to continue.

I. BACKGROUND

1. What is the name of your Call Center?
    a. _________[Free text]__________

2. Which modes of communication are supported by your Call Center? *Select all that apply.*
    a. Telephone
    b. Text/SMS
    c. Online chat
    d. Video call
    b. Other: [Write in]

3. Is your Call Center part of the 988 Lifeline network? *Select all that apply.*
    a. Yes, for calls
    b. Yes, for texts
    c. Yes, for chats
    d. No [make answer exclusive]

4. [If a, b, or c selected on Q3] Do you answer only 988 contacts or multiple lines (for example 211, 311, or a local crisis/suicide prevention number)?
    a. Only 988
    b. Multiple lines

5. [if b selected on Q4] In the past year, about what percent of your contacts were reached through 988?
    a. [Enter percent]
    b. I don't know

6. [if no (d) selected or no response on Q3, i.e., not at 988 center] Does your Call Center <u>operate 24/7</u>: that is, 24 hours per day, 7 days per week?
    a. Yes
    b. No → How many days per week do you operate? *Enter number*.
        → How many hours per day do you operate on open days? *Enter number*.

II. STAFFING

*Next, we are asking questions related to staffing. Here, we will be talking about **counselors**, by which we mean individuals who answer any contact: calls, texts, or chats. This position may also be known as a "crisis counselor" or "hotline specialist" at your Call Center.*

If you are unsure of exact numbers, don't worry. Just take your best guess!

7. Are counselors paid personnel or volunteers?
    a. Paid personnel
    b. Volunteers or unpaid interns
    c. A combination of paid personnel and volunteers/interns

8. [If c selected on Q7] Giving your best guess, what percent of counselors are currently **<u>paid personnel</u>**?
    a. [Enter percent]
    b. I don't know

9. How many **<u>full-time equivalent</u>** counselors currently work at your Call Center? Include paid staff and volunteers/unpaid inters.
   Note: by full-time equivalent, we mean the "equivalent" of 40-hour work week. For example, 4 people working 20 hours per week and 10 people working 8 hours per week would both add up to 80 hours per week, or 2 full-time equivalents.
    a. [Enter number]
    b. I don't know

10. What is the **<u>ideal number</u>** of additional full-time equivalent counselors needed to fully staff your Call Center?
    a. We are fully staffed

    b. [Enter number]
    c. I don't know

11. We expect that the number of counselors you have working could vary throughout the week. What would you estimate is
    a. The **lowest number** of counselors you have working at one time? *Enter number*.
    b. The **typical number** of counselors you have working at one time? *Enter number*.
    c. The **highest number** of counselors you have working at one time? *Enter number*.

12. [if b selected on Q4] Do counselors **exclusively answer** for 988 or **switch between lines** within one work shift (for example answer a 988 call, then answer a call to your local number, then answer 988)?
    a. Answer only for 988
    b. Switch between numbers
    c. A mix of both (i.e., some counselors answer only for 988, and some counselors switch between numbers)

13. [If a on Q12] Of the counselors working during a typical shift, what percent (0-100%) would you say are:
    a. Only answering 988 calls? *Enter percent*.
    b. Only answering calls to your local number? *Enter percent*.

14. [if more than one mode selected on Q2] Do counselors **exclusively answer** one mode of contact (for example, only answer calls or only answer texts), or **switch between modes** within one work shift (for example answer a chat, then answer a text, then answer a call)?
    a. Answer one mode
    b. Switch between modes

15. [If a on Q14] Of the counselors working during a typical shift, what percent (0-100%) would you say are:
    a. [Display if 2a is selected] Only answering calls? *Enter percent*.
    b. [Display if 2b is selected] Only answering texts? *Enter percent*.
    c. [Display if 2c is selected] Only answering chats? *Enter percent*.

16. Are counselors required to work in-person from your Call Center at all times, allowed to work remotely at all times, or allowed a hybrid schedule (home part-time, Call Center part-time)?
    a. Counselors must work at all times in-person from the Call Center
    b. Counselors must work at least part of the time in-person from the Call Center
    c. Counselors are allowed to work remotely 100% of the time
    d. I don't know

17. For each of the following items, please indicate the level of difficulty your call center experiences with regard to staffing.

© 2026 Matthews S et al. *JAMA Network Open.*

Category	Not Difficult	Slightly Difficult	Fairly Difficult	Difficult	Very Difficult
Staffing an adequate number of responders for the volume of contacts received					
Acquiring funding or resources to hire staff					
Recruiting responders					
Retaining responders					

III. 911 INTEROPERABILITY

Next, we ask questions related to interoperability with 911 (i.e., the ability of your Call Center to transfer contacts to 911 and/or receive call transfers from 911).

If you are unsure of exact numbers, don't worry. Just take your best guess!

18. Does your Call Center transfer contacts to 911? *Select all that apply.*
    a. Yes, for calls
    b. Yes, for texts
    c. Yes, for chats
    d. Yes, for videophone
    e. No [make answer exclusive]
    f. I don't know [make answer exclusive]

19. [If yes [a-d] selected on Q18] Approximately how many of your contacts are **transferred to 911**?
    a. Fewer than 1 in 1,000 calls (extremely rare)
    b. Between 1 in 1,000 and 1 in 100 (infrequent but happens)
    c. Between 1 in 100 and 1 in 20 calls (happens with some regularity)
    d. Between 1 in 20 and 1 in 10 calls (happens regularly)
    e. More often than 1 in 10 calls (happens often)

20. [If yes selected on Q19] For contacts transferred to 911, does your organization collect any of the following information about caller outcomes?

	Yes	No	I don't know
Behavioral health outcomes (e.g., levels of distress, suicide risk)			
Referrals to community-based services			
Suicide deaths and attempts			
Psychiatric holds			
Transfers to an emergency department			
Transfers to a psychiatric facility (e.g., crisis stabilization unit, hospital)			
Criminal justice outcomes (e.g., arrests, incarcerations)			

21. Does 911 transfer contacts to your Call Center? *Select all that apply.*
    a. Yes, for calls

© 2026 Matthews S et al. *JAMA Network Open.*

   b. Yes, for texts
   c. No [make answer exclusive]
   d. I don’t know [make answer exclusive]

22. [If yes [a-b] selected on Q21] Approximately how many of your contacts are **transferred from 911 to you**?
   a. Fewer than 1 in 1,000 calls (extremely rare)
   b. Between 1 in 1,000 and 1 in 100 (infrequent but happens)
   c. Between 1 in 100 and 1 in 20 calls (happens with some regularity)
   d. Between 1 in 20 and 1 in 10 calls (happens regularly)
   e. More often than 1 in 10 calls (happens often)

IV. MOBILE CRISIS SERVICES

23. Does the region you serve (e.g., state, county, or counties) have a **mobile crisis response team** available (i.e., mental health professionals to respond to behavioral health crises instead of law enforcement)?
   a. Yes, for all areas
   b. Yes, for some areas
   c. No
   d. I don’t know

24. [If a or b selected on Q23] Does your call center **directly dispatch** the mobile crisis response team (i.e., responders have a direct contact and ability to send the mobile crisis team)?
   a. Yes
   b. No
   c. I don’t know

25. [If yes selected on Q23] Is mobile crisis response **available 24/7**?
   a. Yes
   b. No
   c. I don’t know

26. [If yes selected on Q23] For contacts that receive a mobile crisis response, does your organization collect any of the following information about caller outcomes?

	Yes	No	I don’t know
Behavioral health outcomes (e.g., levels of distress, suicide risk)			
Referrals to community-based services			
Suicide deaths and attempts			
Psychiatric holds			
Transfers to an emergency department			
Transfers to a psychiatric facility (e.g., crisis stabilization unit, hospital)			

© 2026 Matthews S et al. *JAMA Network Open.*

Criminal justice outcomes (e.g., arrests, incarcerations)			

V. COMMUNITY-BASED SERVICES

27. Can your Call Center make direct transfers (i.e., a real-time warm handoff) to **community-based service providers** such as psychiatric health facilities or out-patient mental health providers?
    a. Yes, for all contacts
    b. Yes, for some contacts
    c. No
    d. I don't know

28. Can your Call Center make direct transfers (i.e., a real-time warm handoff) to **clinicians/therapists** via telehealth?
    a. Yes, for all contacts
    b. Yes, for some contacts
    c. No
    d. I don't know

29. Can your Call Center **schedule intake and outpatient appointments** on behalf of callers?
    a. Yes, for all contacts
    b. Yes, for some contacts
    c. No
    d. I don't know

CONCLUSIONS

30. If you are open to participating in a brief follow-up interview to discuss call center staffing in more detail, please enter your first and last name (this will be kept confidential): ____________________________

31. If you would like to receive a $50 Amazon gift card for completing this survey, please provide your email address: ______________________________

32. What is your title/role at your Call Center (e.g., Director, Responder)? ___________________

33. To the best of your knowledge, what state or states does your Call Center primarily serve when people dial 988 or contact your Call Center directly (e.g., TX, IL, MA)? ______________

34. If there is anything you would like to comment on regarding this survey, please enter it here: ____________________________

THANK YOU FOR YOUR TIME!

© 2026 Matthews S et al. *JAMA Network Open.*

If you have questions or concerns about your rights as a participant in this survey, you may contact RAND’s Human Subjects Protection Committee toll-free at (866) 697-5620 or by emailing hspcinfo@rand.org.

© 2026 Matthews S et al. *JAMA Network Open.*
