# Supplementary material for: Workforce and Staffing at 988 Suicide & Crisis Lifeline Centers
Source: JAMA Netw Open. 2026 May 5;9(5):e2610789. doi: 10.1001/jamanetworkopen.2026.10789 (PMC13147195; doi:10.1001/jamanetworkopen.2026.10789)
Supplement: Supplement 2. — Data Sharing Statement [file jamanetwopen-e2610789-s002.pdf]

Data Sharing Statement

Matthews. Workforce and Staffing at 988 Suicide & Crisis Lifeline Centers. *JAMA Netw Open*. Published May 05, 2026. doi:10.1001/jamanetworkopen.2026.10789

Data
Data available: Yes
Data types: Deidentified participant data
How to access data: smatthew@rand.org
When available: With publication

Supporting Documents
Document types: None

Additional Information
Who can access the data: Researchers whose proposed use of the data has been approved
Types of analyses: For any purpose
Mechanisms of data availability: With signed data access agreement
